# Supplementary material for: Analysis of PPARγ Signaling Activity in Psoriasis
Source: Int J Mol Sci. 2021 Aug 10;22(16):8603. doi: 10.3390/ijms22168603 (PMC8395241; doi:10.3390/ijms22168603)
Supplement: Supplementary file 1 [file ijms-22-08603-s001.zip › Supplemental materials_Analysis of PPARg signaling activity in psoriasis/Pathway models/Models images and html files/Differentiation of psoriatic T cells/6313.html]

IL1R1


# Protein IL1R1

|  |  |
| --- | --- |
| URN | urn:agi-llid:3554 |
| Total Entities | 0 |
| Connectivity | 1333 |
| Name | IL1R1 |
| Description | interleukin 1 receptor, type I |
| Notes | This gene encodes a cytokine receptor that belongs to the interleukin-1 receptor family. The encoded protein is a receptor for interleukin-1 alpha, interleukin-1 beta, and interleukin-1 receptor antagonist. It is an important mediator involved in many cytokine-induced immune and inflammatory responses. This gene is located in a cluster of related cytokine receptor genes on chromosome 2q12. [provided by RefSeq, Dec 2013] |
| Primary Cell Localization | Plasma membrane |
| Class | Receptor |

---

|  |  |
| --- | --- |
| Pathway | Osteoarthritis |
|  | Th17-Cell Differentiation |
|  | Bradykinin Effects in Inflammation |
|  | Toll-like Receptor Independent Sterile Inflammation |
|  | Mast-Cell Activation without Degranulation |
|  | Mast-Cell Activation without Degranulation through IL1R1 and TLR Signaling |
|  | anti-Inflammatory Function of Macrophage M2 Lineage |
|  | Negative Acute Phase Proteins Synthesis |
|  | Positive Acute Phase Proteins Synthesis |
|  | Vascular Endothelial Cell Activation by Cytokines |
|  | IL1B Expression Targets -> Nociception |
|  | CD8+ Naive T-cell -> CD4+ Naive T-cell Surface Expression Markers |
|  | Megakaryocyte -> Platelet Precursor Cell Surface Expression Markers |
|  | T-lymphoid precursor cell -> CD8+ Naive T-cell Surface Expression Markers |
|  | Lymphocyte Mediated Myocardial Injury in Myocarditis |
|  | Proteins Involved in Myocarditis |
|  | Inflammatory Reaction in Acne Vulgaris |
|  | Keratinocyte Proliferation in Acne Vulgaris |
|  | T-Cells Differentiation Block in Psoriasis |
|  | Th17-Cell Function in Systemic Lupus Erythematosus |
|  | Vitiligo |
|  | T-Cell Cytotoxic Response against Melanocytes in Vitiligo |
|  | Toll-like Receptors in beta-Cell |
|  | beta-Cell Destruction through Cytokines in Diabetes Mellitus |
|  | beta-Cell Destruction in Diabetes Mellitus Type 1 |
|  | Nitric Oxide Effects on beta-Cell |
|  | CD4+T-Cell Response in Celiac Disease |
|  | Leukocyte Migration toward the Endothelial Cell in Ulcerative Colitis |
|  | TSLP Signaling in Bronchial Epithelial Cell in Asthma |
|  | Th17-Cell Differentiation in Asthma |
|  | CFTR Facilitates Oxidative Stress in Airway Epithelium in CF |
|  | CFTR Expression in Epithelial Cells (Class I Mutations) |
|  | Osteoclast Activation in Gout |
|  | Neutrophil Recruitment in Sinovium in Gout |
|  | TNF and IL1B Induce Metalloproteinase Synthesis in Osteoarthritis |
|  | IL1B Induced Arthralgia |
|  | Th17-Cell and Th1 Immune Responsein Psoriatic Arthritis |
|  | Osteoclast Activation in Psoriatic Arthritis |
|  | Synovial Fibroblast Activation in Psoriatic Arthritis |
|  | Osteoclast Activation in Rheumatoid Arthritis |
|  | Synovial Fibroblast Activation by Citokines in Rheumatoid Arthritis |
|  | Autocrine Cytokine/Chemokine Loops in Systemic Scleroderma |
|  | Th17-Cell Numbers Reduction in HIV |
|  | IL1 Receptor Antagonist (IL1RN) Deficiency (DIRA) |
|  | IL36 Receptor Antagonist (IL1F5) Deficiency (DITRA) |
|  | HMGB1 and IL1B in Neuroinflammation |
|  | HMGB1 and IL1B in Neuronal Hyperexcitation |
|  | Proteins Involved in Endometriosis |
|  | Subepithelial Fibroblasts in Middle Ear Cholesteatoma |
|  | Cytokines Trigger Otitis Media |
|  | Alveolar Macrophages Dysfunction in COPD |
|  | Mucin Hyperproduction in Goblet and Mucous Cells |
|  | Endothelial Cell Dysfunction in Glomerulonephritis |
|  | Endothelial Cell Dysfunction in Pyelonephritis |
|  | Elevated Receptors -> Expression Targets in Blood vessel |
|  | Elevated Receptors -> Expression Targets in Blood Vessel |
|  | Elevated Receptors -> Expression Targets in Small Intestine |
|  | NFKB Canonical Signaling Activation in Cancer |
|  | STAT3 and NFkB Activate Inflammation-Induced Tumorigenesis |
|  | NFKB Signaling Activation by Blocking of Tumor Suppressors |
|  | IDO1 in Cancer Immune Escape |
|  | IGF1 -> CEBPA/FOXO1A Expression Targets |
|  | IL1R -> STAT3 Signaling |
|  | IL1R -> NF-kB Signaling |
|  | IL6 Expression Targets |
|  | Interleukin Receptors -> Expression Targets in Lymphoid System and Blood |
|  | Elevated Receptors -> Expression Targets in Nerve Tissue |
|  | NF-kB Canonical Signaling |
|  | TLR4 -> AP-1/EGR1/HIF1A Expression Targets |
|  | IL1B Expression Targets |
|  | IL1A Expression Targets |
|  | PPAR Psoriasis |
|  | Model of PPARG signaling in psoriasis |
|  | 1\_Inflammation in keratinocytes in the pathology of acne |
|  | 1\_Differentiation of psoriatic T cells |
|  | 1\_TNF and IL1B provoke ECM degradation in osteoarthritis |
|  | 2\_Chondrocyte death in osteoarthritis |
|  | 2\_The role of endothelial cells in pyelonephritis |
|  | 1\_Insufficient activation of immune response in the middle ear epithelium cells in otitis media |
|  | 2\_Pathogens stimulate mucins expression in the middle ear |
|  | 3\_Role of alveolar macrophages in pulmonary tissue destruction in COPD |
|  | 3\_1\_Mucus accumulation in asthma |
|  | 4\_Mucin hyperproduction in goblet and mucous cells in COPD |
|  | 3\_Leukocyte migration toward the endothelial cells in the intestine microvasculature |
|  | 1\_CFTR expression and degradation in epithelial cells in cystic fibrosis (class I-II mutations) |
|  | 1\_Antigen-presenting cells promote islet dysfunction and immune system activation in T1D |
|  | 3\_Islet beta-cell destruction in T1D |
|  | Differentiation of psoriatic T cells |

---

|  |  |
| --- | --- |
| MedScan ID | 3554 |

---

|  |  |
| --- | --- |
| LocusLink ID | 3554 |
|  | 16177 |
|  | 25663 |

---

|  |  |
| --- | --- |
| Alias | OTTHUMP00000161344 |
|  | interleukin IL1R\_HUMAN |
|  | D2S1473 |
|  | MGC129154 |
|  | IL1 Receptor alpha |
|  | interleukin 1 receptor, type I |
|  | IL1R I |
|  | OTTHUMP00000203891 |
|  | interleukin 1 receptor 1 |
|  | interleukin IL1 receptor alpha |
|  | CD121a antigen |
|  | IL-iR |
|  | interleukin 1 receptors type I |
|  | CD121a |
|  | interleukin IL1 receptor 1 |
|  | IL1 receptor I |
|  | interleukin 1 beta receptor type 1 short transmembrane form |
|  | IL-1R-alpha |
|  | interleukin IL1 receptor alpha, type I |
|  | IL1 receptor 1 |
|  | IL-1RT1 |
|  | OTTHUMP00000203889 |
|  | IL1R1 |
|  | interleukin IL1R I |
|  | interleukin 1 receptors type 1 |
|  | interleukin 1 receptor alpha, type 1 |
|  | OTTHUMP00000203888 |
|  | OTTHUMP00000203887 |
|  | interleukin IL1 receptor I |
|  | interleukin 1 receptor alpha, type I |
|  | IL1R\_HUMAN |
|  | CD121 antigen-like family member A |
|  | interleukin IL1 receptor alpha, type 1 |
|  | interleukin 1 bete receptor type 1 long transmembrane form |
|  | Antigen CD121a |
|  | OTTHUMP00000207977 |
|  | interleukin IL-1R-alpha |
|  | interleukin-1 receptor alpha |
|  | interleukin IL1R1 |
|  | interleukin 1 beta receptor type 1 soluble form |
|  | interleukin 1 beta receptor type 1 long transmembrane form |
|  | P80 |
|  | IL1R |
|  | IL1RA |
|  | IL-1R-1 |
|  | IL-1RT-1 |
|  | interleukin-1 receptor type I |
|  | interleukin-1 receptor type 1 |
|  | CD121b |
|  | IL-1R1 |
|  | Il1r-1 |
|  | IL-1 receptor alpha chain |
|  | IL1RT1 |
|  | interleukin receptor 1 |

---

|  |  |
| --- | --- |
| GO ID | 0019966 |
|  | 0004908 |
|  | 0004909 |
|  | 0005161 |
|  | 0002020 |
|  | 0004888 |
|  | 0007166 |
|  | 0019221 |
|  | 0006955 |
|  | 0006954 |
|  | 0070498 |
|  | 2000556 |
|  | 0032729 |
|  | 2000661 |
|  | 2000391 |
|  | 0050727 |
|  | 0070555 |
|  | 0009897 |
|  | 0005576 |
|  | 0005887 |
|  | 0016020 |
|  | 0005886 |
|  | 0035255 |
|  | 0010286 |
|  | 2001224 |
|  | 0009314 |
|  | 0007165 |
|  | 0030424 |
|  | 0009986 |
|  | 0005615 |
|  | 0016021 |
|  | 0005634 |
|  | 0014069 |
|  | 0032991 |
|  | 0071333 |
|  | 0030728 |
|  | 0070849 |
|  | 0071731 |
|  | 1990834 |
|  | 0071559 |
|  | 0004871 |
|  | 0043234 |
|  | 0045087 |
|  | 0005622 |
|  | 0031224 |
|  | 0004872 |

---

|  |  |
| --- | --- |
| KEGG ID | hsa:3554 |
|  | mmu:16177 |
|  | rno:25663 |

---

|  |  |
| --- | --- |
| Organism | Homo sapiens {Organism urn:agi-taxid:9606} |
|  | Mus musculus {Organism urn:agi-taxid:10090} |
|  | Rattus norvegicus {Organism urn:agi-taxid:10116} |
|  | Mus musculus |
|  | Homo sapiens |
|  | Rattus norvegicus |

---

|  |  |
| --- | --- |
| Mouse chromosome position | 1 18.81 cM |
|  | 1 19.5 cM |

---

|  |  |
| --- | --- |
| OMIM ID | 147810 |

---

|  |  |
| --- | --- |
| Rat chromosome position | 9q22 |
|  | 9q21 |

---

|  |  |
| --- | --- |
| Hugo ID | 5993 |
|  | HGNC:5993 |

---

|  |  |
| --- | --- |
| Human chromosome position | 2q11.2-q12.1 |
|  | 2q12 |

---

|  |  |
| --- | --- |
| Swiss-Prot Accession | P14778 |
|  | B8ZZW4 |
|  | P14778.1 |
|  | Q8C6P3 |
|  | Q8C833 |
|  | P13504 |
|  | Q32MH0 |
|  | P13504.1 |
|  | Q05KR1 |
|  | Q05KR0 |
|  | A0A0G2K6N4 |
|  | Q02955.1 |
|  | Q587I7 |
|  | Q02955 |
|  | Q6NWP5 |
|  | Q6NWP6 |
|  | Q05KR2 |
|  | Q62692 |
|  | Q76LI4 |

---

|  |  |
| --- | --- |
| PIR ID | A36187 |
|  | A32604 |

---

|  |  |
| --- | --- |
| GenBank ID | NC\_000002 |
|  | XM\_011511117 |
|  | XP\_011509419 |
|  | NM\_001320978 |
|  | NP\_001307907 |
|  | NM\_001288706 |
|  | NP\_001275635 |
|  | XM\_011511115 |
|  | XP\_011509417 |
|  | NM\_001320980 |
|  | NP\_001307909 |
|  | NM\_001320986 |
|  | NP\_001307915 |
|  | XM\_011511116 |
|  | XP\_011509418 |
|  | XM\_017003989 |
|  | XP\_016859478 |
|  | NM\_001320981 |
|  | NP\_001307910 |
|  | XM\_005263930 |
|  | XP\_005263987 |
|  | NM\_001320982 |
|  | NP\_001307911 |
|  | NM\_000877 |
|  | NP\_000868 |
|  | XM\_005263934 |
|  | XP\_005263991 |
|  | XM\_011511118 |
|  | XP\_011509420 |
|  | NM\_001320985 |
|  | NP\_001307914 |
|  | NM\_001320984 |
|  | NP\_001307913 |
|  | NM\_001320983 |
|  | NP\_001307912 |
|  | AC007271 |
|  | AAX81988 |
|  | AF172151 |
|  | AF190052 |
|  | AF366364 |
|  | AF531102 |
|  | AAM88423 |
|  | AH008153 |
|  | AX530423 |
|  | CAD57171 |
|  | CH471127 |
|  | EAX01799 |
|  | JA738563 |
|  | CCF76934 |
|  | KF456690 |
|  | S81089 |
|  | AK026803 |
|  | AK314433 |
|  | AL548240 |
|  | AU136426 |
|  | BC067506 |
|  | AAH67506 |
|  | BC067507 |
|  | AAH67507 |
|  | BC067508 |
|  | AAH67508 |
|  | BC075062 |
|  | AAH75062 |
|  | BC075063 |
|  | AAH75063 |
|  | BE144272 |
|  | BQ021163 |
|  | BX472731 |
|  | CA421991 |
|  | CB240976 |
|  | CD013912 |
|  | CD013913 |
|  | CD014140 |
|  | DA333942 |
|  | DA861783 |
|  | DB226934 |
|  | HY092388 |
|  | M27492 |
|  | AAA59137 |
|  | X16896 |
|  | CAA34773 |
|  | P14778 |
|  | NC\_000067 |
|  | XM\_006495713 |
|  | XP\_006495776 |
|  | XM\_006495719 |
|  | XP\_006495782 |
|  | XM\_006495722 |
|  | XP\_006495785 |
|  | NM\_008362 |
|  | NP\_032388 |
|  | XM\_017317595 |
|  | XP\_017173084 |
|  | XM\_017317613 |
|  | XP\_017173102 |
|  | XM\_006495723 |
|  | XP\_006495786 |
|  | XM\_006495721 |
|  | XP\_006495784 |
|  | XM\_011238436 |
|  | XP\_011236738 |
|  | XM\_006495715 |
|  | XP\_006495778 |
|  | XM\_006495720 |
|  | XP\_006495783 |
|  | XM\_006495714 |
|  | XP\_006495777 |
|  | NM\_001123382 |
|  | NP\_001116854 |
|  | XM\_006495717 |
|  | XP\_006495780 |
|  | AC135085 |
|  | AC167168 |
|  | CH466536 |
|  | EDL14572 |
|  | AK048550 |
|  | BAC33372 |
|  | AK054137 |
|  | BAC35666 |
|  | AK166301 |
|  | BC109135 |
|  | AAI09136 |
|  | M20658 |
|  | AAA39279 |
|  | P13504 |
|  | NC\_005108 |
|  | XM\_006244753 |
|  | XP\_006244815 |
|  | XM\_006244747 |
|  | XP\_006244809 |
|  | XM\_006244752 |
|  | XP\_006244814 |
|  | XM\_006244748 |
|  | XP\_006244810 |
|  | XM\_006244750 |
|  | XP\_006244812 |
|  | XM\_006244746 |
|  | XP\_006244808 |
|  | XM\_006244754 |
|  | XP\_006244816 |
|  | XM\_006244749 |
|  | XP\_006244811 |
|  | XM\_008766993 |
|  | XP\_008765215 |
|  | XM\_006244751 |
|  | XP\_006244813 |
|  | NM\_013123 |
|  | NP\_037255 |
|  | AC\_000077 |
|  | AABR07067457 |
|  | AABR07067458 |
|  | AAHX01059540 |
|  | AAHX01059541 |
|  | AAHX01059542 |
|  | AAHX01059543 |
|  | CH473965 |
|  | EDL99179 |
|  | EDL99180 |
|  | EDL99181 |
|  | AB079118 |
|  | BAD15353 |
|  | AB112436 |
|  | BAF34663 |
|  | AB112437 |
|  | BAF34664 |
|  | AB112438 |
|  | BAF34665 |
|  | M95578 |
|  | AAA16196 |
|  | U14010 |
|  | AAA50243 |
|  | Q02955 |
|  | AF146426 |
|  | AF146427 |
|  | XM\_005263932 |
|  | XP\_005263989 |
|  | XM\_005263929 |
|  | XP\_005263986 |
|  | XM\_005263931 |
|  | XP\_005263988 |
|  | XM\_011511114 |
|  | XP\_011509416 |
|  | XM\_011511120 |
|  | XP\_011509422 |
|  | XM\_011511119 |
|  | XP\_011509421 |
|  | XM\_005263933 |
|  | XP\_005263990 |
|  | NC\_018913 |
|  | AMYH02004435 |
|  | XM\_006495716 |
|  | XP\_006495779 |
|  | XM\_006495718 |
|  | XP\_006495781 |
|  | AC\_000023 |
|  | AAHY01005650 |
|  | AAHY01005651 |
|  | AAHY01005652 |
|  | AAHY01005653 |
|  | AAHY01005654 |
|  | AAHY01005655 |
|  | AAHY01005656 |
|  | XR\_244889 |
|  | AC\_000134 |
|  | ABBA01073900 |
|  | NT\_022171 |
|  | NW\_001838818 |
|  | NW\_928052 |
|  | AC\_000045 |
|  | CR595183 |
|  | CR621155 |
|  | Q6NWP5 |
|  | Q6NWP6 |
|  | NT\_039170 |
|  | NW\_001030649 |
|  | Q32MH0 |
|  | Q8C6P3 |
|  | Q8C833 |
|  | NW\_047814 |
|  | NW\_001084882 |
|  | Q05KR0 |
|  | Q05KR1 |
|  | Q05KR2 |
|  | Q62692 |
|  | Q76LI4 |

---

|  |  |
| --- | --- |
| Swiss-Prot ID | IL1R1\_HUMAN |
|  | IL1R1\_MOUSE |
|  | Q32MH0\_MOUSE |
|  | Q05KR1\_RAT |
|  | Q8C6P3\_MOUSE |
|  | Q8C833\_MOUSE |
|  | Q587I7\_HUMAN |

---

|  |  |
| --- | --- |
| Cell Localization | Membrane |
|  | Cell membrane |
|  | Secreted |
|  | Plasma membrane |

---

|  |  |
| --- | --- |
| Ensembl ID | ENSG00000115594 |
|  | ENSP00000386380.1 |
|  | ENST00000410023.6 |
|  | ENSP00000386776.1 |
|  | ENST00000409929.5 |
|  | ENSP00000386555.1 |
|  | ENST00000409589.5 |
|  | ENSMUSG00000026072 |
|  | ENSMUSP00000110443.1 |
|  | ENSMUST00000114795.2 |
|  | ENSMUSP00000027241.4 |
|  | ENSMUST00000027241.10 |
|  | ENSRNOG00000014504 |
|  | ENSRNOP00000072248.1 |
|  | ENSRNOT00000082408.1 |
|  | ENSRNOP00000073880.1 |
|  | ENSRNOT00000082146.1 |
|  | ENSRNOP00000019673.3 |
|  | ENSRNOT00000019673.4 |
|  | ENSRNOP00000075417.1 |
|  | ENSRNOT00000087112.1 |
|  | ENST00000410023.5 |
|  | ENSP00000386380 |
|  | ENST00000410023 |
|  | ENSP00000386776 |
|  | ENST00000409929 |
|  | ENSMUSP00000110443 |
|  | ENSMUST00000114795 |
|  | ENSMUSP00000027241 |
|  | ENSMUST00000027241 |
|  | ENSRNOP00000072248 |
|  | ENSRNOT00000082408 |
|  | ENSRNOP00000073880 |
|  | ENSRNOT00000082146 |
|  | ENSRNOP00000019673 |
|  | ENSRNOT00000019673 |
|  | ENSRNOP00000075417 |
|  | ENSRNOT00000087112 |

---

|  |  |
| --- | --- |
| MGI ID | MGI:96545 |
|  | 96545 |

---

|  |  |
| --- | --- |
| RGD ID | 2892 |

---

|  |  |
| --- | --- |
| TCDB ID | 8.A.23.1.11 |

---

|  |  |
| --- | --- |
| Unigene ID | Rn.9758 |
|  | Hs.701982 |
|  | Mm.896 |

---

|  |  |
| --- | --- |
| Homologene ID | 677 |

---

|  |  |
| --- | --- |
| Shape | Stick-vertex |

---

|  |  |
| --- | --- |
| IPI ID | IPI00122581 |
|  | IPI00027508 |
|  | IPI00231728 |
|  | IPI00875537 |

---
